# Treatment of Children With Attention-Deficit/Hyperactivity Disorder (ADHD) and Irritability: Results From the Multimodal Treatment Study of Children With ADHD (MTA)

**DOI:** 10.1016/j.jaac.2014.10.006

**Published:** 2015-01

**Authors:** Lorena Fernández de la Cruz, Emily Simonoff, James J. McGough, Jeffrey M. Halperin, L. Eugene Arnold, Argyris Stringaris

**Affiliations:** a Institute of Psychiatry, King’s College London, UK; b Semel Institute for Neuroscience and Human Behavior at the University of California, Los Angeles, USA; c Queens College, City University of New York, New York City, USA; d Nisonger Center, The Ohio State University, Columbus, OH, USA

**Keywords:** irritability, attention-deficit/hyperactivity disorder, oppositional defiant disorder, treatment outcomes

## Abstract

**Objective:**

Clinically impairing irritability affects 25% to 45% of children with attention-deficit/hyperactivity disorder (ADHD); yet, we know little about what interventions are effective in treating children with ADHD and co-occurring irritability. We used data from the Multimodal Treatment Study of Children With ADHD (MTA) to address 3 aims: to establish whether irritability in children with ADHD can be distinguished from other symptoms of oppositional defiant disorder (ODD); to examine whether ADHD treatment is effective in treating irritability; and to examine how irritability influences ADHD treatment outcomes.

**Method:**

Secondary analyses of data from the MTA included multivariate analyses, and intent-to-treat random-effects regression models were used.

**Results:**

Irritability was separable from other ODD symptoms. For treating irritability, systematic stimulant treatment was superior to behavioral management but not to routine community care; a combination of stimulants and behavioral treatment was superior to community care and to behavioral treatment alone, but not to medication alone. Irritability did not moderate the impact of treatment on parent- and teacher-reported ADHD symptoms in any of the 4 treatment groups.

**Conclusion:**

Treatments targeting ADHD symptoms are helpful for improving irritability in children with ADHD. Moreover, irritability does not appear to influence the response to treatment of ADHD.

**Clinical trial registration information:**

Multimodal Treatment Study of Children With Attention Deficit and Hyperactivity Disorder (MTA); http://www.clinicaltrials.gov; NCT00000388.

Clinically impairing irritability affects 25% to 45% of children with attention-deficit/hyperactivity disorder (ADHD)^1^; yet, the evidence base for treatment selection in the presence of irritability remains thin. This article addresses this knowledge gap by analyzing data from the Multimodal Treatment Study of Children With ADHD (MTA), a large randomized trial comparing various treatment modalities among children with ADHD.^2^

ADHD is among the most common child psychiatric disorders worldwide.^3^ It is defined by chronic, pervasive, and impairing symptoms of inattention and hyperactivity/impulsivity.^4^ Although irritability, defined by temper outbursts and proneness to anger,^5^ is not a diagnostic criterion for ADHD, it is a common presentation in this clinical group^6,7^ and is listed under the associated features of ADHD in the *DSM*.^4^ In an epidemiological study, 38% of children with ADHD had irritable mood, nearly 10-fold higher over general population rates.^8^ This raises the question of how best to treat the subgroup of children with ADHD and irritability.

One approach to this question is to consider irritability as one of the manifestations of behavior problems that are typical of ADHD. Indeed, irritability is characteristic of children with oppositional defiant disorder (ODD), which is highly comorbid with ADHD.^9^ Substantial evidence suggests that stimulant treatment also reduces ODD symptoms in those with ADHD^2,10,11^ and that parenting interventions may also be useful.^12^ However, this is an assumption that needs to be explicitly tested, as mounting evidence indicates that irritable mood is distinct from other, typically headstrong, behaviors characteristic of ODD. This distinction is reflected in the *DSM-5*^4^ and is based on research showing that irritability is separable from headstrong behaviors (e.g., argumentativeness, noncompliance, and rule breaking) by virtue of its multivariate structure,^13^ longitudinal course,^14,15^ external predictions,^16^ and genetic associations.^17^ In particular, irritability predicts subsequent depression and generalized anxiety, whereas headstrong behaviors predict subsequent delinquent behaviors.^16^ If irritability is clinically and etiologically distinct from other behavior problems, irritability in ADHD may also require distinct treatment compared to headstrong behaviors. However, there is little research on the distinctions between these 2 groups of symptoms in children with ADHD^13^ or evidence about how best to treat irritable children who have ADHD.

Here we use data from the MTA to address these questions by examining 3 aims. First, we wanted to establish the robustness and clinical relevance of irritability in the MTA. In particular, it is important to know whether irritability in children with ADHD can be distinguished from other typical symptoms of oppositionality, namely, headstrong symptoms. We hypothesized the following: that irritability would be separable from headstrong behaviors in multivariate analyses; that irritability would have different external correlates/consequences (building upon prior investigations,^14^ we expected that irritability would significantly differentially predict internalizing symptoms and disorders such as depression and anxiety, whereas the headstrong dimension would differentially predict conduct problems, such as conduct disorder [CD]); that irritability would show sufficient longitudinal continuity in children with ADHD such that it could be differentiated from headstrong symptoms; and that irritable and headstrong behaviors each would contribute independently to impairment.

Our second aim was to ascertain how irritability in ADHD responds to treatment with stimulants and/or behavioral therapy. Clinical experience and prior results from randomized controlled treatment studies in children^18-20^ suggest that stimulant treatment may be useful to treat irritability in ADHD and should be considered as a first-line treatment^1^; however, the evidence is somewhat mixed. Two randomized controlled trials comparing amphetamine and placebo found no beneficial effect of the medication on a broad range of emotional problems, and some studies have found that amphetamine preparations increase irritability and lability.^21^ On the other hand, it has recently been shown that children with ADHD and behavior problems that are closely linked to irritability probably respond to behavioral treatment.^22,23^ Similarly, a recent meta-analysis of randomized controlled trials^12^ has provided evidence from blinded outcomes that behavioral interventions improve parenting and reduce childhood conduct problems in ADHD. To address this matter, we examine 2 competing hypotheses: 1 hypothesis based on the preliminary findings above that suggest stimulant treatment could be helpful; and the other hypothesis, which is that because irritability can be a component of behavior problems such as ODD, it may respond well to behavioral treatment.

The third aim was to establish whether the response to treatment of children with ADHD and irritability differed from that of children without irritability. There is surprisingly little research in this area, although a previous study using MTA data indicated that symptoms of mania do not influence the treatment response to methylphenidate or its side effect profile in children with ADHD.^24^ However, this investigation used the 1-month methylphenidate titration trial subset of the MTA and therefore did not include the comparisons with the behavioral interventions nor the treatment outcome after 14 months. A subsequent study by Galanter and colleagues, also using MTA data, showed that children with manic symptoms, as defined using the Child Behavior Checklist (CBCL) dysregulation profile, suffered more morbidity at study onset, yet they also responded to standard ADHD treatment without suffering more side effects compared to children without manic symptoms.^25^ This suggests that standard treatment may benefit severely affected children with ADHD; however, the inclusion of anxiety/depression, aggression, and attention in the definition of manic symptoms makes it difficult to estimate the effects of treatment on irritability and on how the presence of irritability may moderate treatment response in children with ADHD. This is particularly important given the separability of irritability from other behavior problems. Hence, in this study, we tested whether the response to MTA treatments, including medication, behavioral treatment, and the combination, varied according to the level of irritability. Our expectation was that while irritable children would show higher levels of ADHD symptoms, they would respond similarly to children low on irritability. In particular, we expect that the previously demonstrated superiority of the medication management over the community comparison and the behavioral treatment arms in the MTA^2^ would remain even when accounting for levels of irritability.

## Method

### Participants and Procedure

A total of 579 children meeting diagnostic criteria for ADHD Combined Type were recruited from 6 different US sites and randomly assigned to 1 of the following 4 groups: medication management (“MedMgt”; n = 144; 24.9%); behavioral treatment (“Beh”; n = 144; 24.9%); combined treatment (“Comb”; n = 145; 25.0%); or treatment-as-usual community comparison (“CC”; n = 146; 25.2%). The first 3 groups were treated for 14 months in specified protocols. Briefly, MedMgt consisted of a 1-month double-blind titration with methylphenidate for best dose, progressing to an open titration with other drugs, such as d-amphetamine, pemoline, or imipramine if methylphenidate was unsatisfactory. Beh consisted of intense, multi-component individual and group parent training; teacher consultation; a child-directed, 8-week, full-time summer treatment program; and use of a 12-week, half-time classroom behavioral specialist. Comb integrated the MedMgt and Beh strategies, with more extensive assistance from the behavioral therapist to assist in medication adjustment and information from the pharmacotherapist to aid in decision making about escalation of behavioral interventions. The fourth group (CC) was referred for community treatment of the parents' choosing. The mean age of the children at baseline was 8.5 years (SD = 0.8 years, range = 7–10 years), and 114 children were female (19.7%). Ethnic composition of the sample included 60.8% of white ethnicity, 19.9% African American, and 19.3% Hispanic, racially mixed, or from other ethnic origins. Treatment groups did not differ significantly at baseline on gender, ethnicity, IQ, comorbidity, Conners Parent and Teacher Rating Scales scores, impairment, and medication for ADHD before the study. The only significant difference was age, although all participants were actually in a tight age range (the youngest were in the behavioral treatment group: mean age = 8.3 years; and the oldest were in the medication management group: mean age = 8.6 years). At least 1 parent and all participants in the original MTA study provided written permission and assent for participation before initiation of any study procedures as approved by each site’s institutional review board. For the present study, all of the participants from the original study were included. Additional details about the sampling and the procedures in the MTA have been widely described elsewhere.^2,26,27^

### Measures

The measures relevant to our study are described below. A comprehensive description of the assessment measures used in the MTA has been described elsewhere.^2,28^

ADHD symptom severity was measured by using the mean score of items 1 to 18 of the parent- and the teacher-reported Swanson, Nolan, and Pelham (SNAP) rating scale,^29^ which includes the inattention and hyperactivity/impulsivity subscales.

In accordance with previous studies,^14,30^ a measure of irritability was generated by adding up the following 3 ODD items on the parent-reported SNAP: “Loses temper”; “Is touchy or easily annoyed by others”; and “Is angry and resentful.” The scores ranged from 0 to 9. In addition, a categorical irritability outcome was generated using a median split into high and low irritability and was used for purposes of illustration in several figures. The irritability measure was based on parent-report, given that parents, as compared to teachers, are rated as more useful informants of children’s emotional problems.^31,32^

A headstrong dimension was generated by adding up 4 ODD items on the parent-reported SNAP: “Argues with adults,” “Actively defies or refuses adult requests or rules,” “Does things deliberately that annoy other people,” and “Blames others for his or her mistakes or misbehavior.” The scores ranged from 0 to 12. The only ODD item not used for either the irritability or the headstrong scales was “Is spiteful or vindictive.”

The CBCL was used to assess general psychopathology. The CBCL is a parent-report checklist mapping onto multiple aspects of psychopathology over a 6-month period.^33^ Children’s global impairment was measured by using the Columbia Impairment Scale–Parent Version (CISP) questionnaire.^34^

### Data Analysis

Data analyses for each specific aim were as follows: 1) To establish whether irritability was independent from other ODD symptoms in the MTA sample, we proceeded in 4 ways. First, we explored differences in the multivariate structure using a confirmatory factor analysis (CFA) comparing 2 models (1 versus 2 factors, namely irritability and headstrong behaviors). Second, we explored the longitudinal continuity of each measure (irritability and headstrong behaviors) using path analysis. Third, we explored whether irritability and headstrong behaviors had different correlates in linear regression models in which the 2 variables were introduced as predictors, Finally, we ran a linear regression in which irritability and headstrong behaviors were predictors of impairment to test whether the 2 dimensions contributed independently to it. This is important because impairment can be independent of symptom severity.^35^ Significant differences between estimates were judged based on nonoverlapping 95% CI. 2) To test the hypothesis that MedMgt would be superior to Beh in treating irritability, we ran an intent-to-treat (ITT) random-effects regression analysis similar to the original primary analyses but with irritability as the outcome, and time (including baseline, 3-month, 9-month, and 14-month assessments) and treatment group as predictors, as well as the interaction time×treatment group. As in the original MTA study, we also tested for site differences and site-by-treatment effects using the interaction site×treatment group. In statistical terms, our hypothesis was that there would be a significant time-by-treatment group interaction, and that by decomposing this interaction we would find that the MedMgt group (as well as the Comb) would be superior to the Beh group. In addition, we calculated the pre–post effect size of each treatment option by using Cohen’s d formula (mean score baseline–mean score at 14 months) / pooled SD. 3) Finally, we tested whether baseline irritability would moderate treatment outcomes, by running an ITT random-effects regression analysis with the severity of the ADHD symptoms as the outcome and, again, time (including baseline, 3-month, 9-month, and 14-month assessments), treatment group, and site as predictors, as well as the interactions irritability×time, irritability×treatment group, time×treatment group, site×treatment group, and irritability×time×treatment group. We also tested whether irritability would differentially affect the response to individual treatments by testing the 3-way interaction of irritability×treatment group×time.

### Ethical Approval

The de-identified MTA dataset (MTA96, Version #1) was provided by the National Institute of Mental Health (NIMH) upon public-access request. The Psychiatry, Nursing, and Midwifery Research Ethics Subcommittee (PNM RESC) at King’s College London approved the secondary analysis of these data (reference PNM/13/14-34).

## Results

The mean score for the whole sample for the parent-reported irritability subscale derived from the ODD items of the SNAP was 4.30 (SD = 2.48) and the median value was 4. Internal consistency was high (Cronbach’s α = .83), and it showed good convergent validity by correlating highly with a scale comprising irritability items from the CBCL (Pearson’s correlation r = 0.66, latent correlation r = 0.72). This measure of irritability derived from the CBCL has also been used in previous research.^17^ The mean score for the whole sample for the parent-reported headstrong behaviors subscale derived from the ODD items of the SNAP was 6.44 (SD = 3.19), and the median value was 7. This variable also showed high internal consistency (Cronbach’s α = 0.83). At baseline, Pearson’s correlations of irritability with ADHD symptoms (r = 0.34; *p* < .001) and with the CISP (r = 0.45; *p* < .001) were in the medium range.

### Aim I: To Establish the Independence of the Irritability Dimension in the MTA Sample

The 2-factor model (irritability versus headstrong behaviors) showed a better fit with the data (AIC = 8,640.38; BIC = 8,765.94) compared to the 1-factor model (AIC = 8,751.85; BIC = 8,873.08), as confirmed by difference testing (value = 69.23, df = 1; *p* ≤ .001).

**Figure S1:**
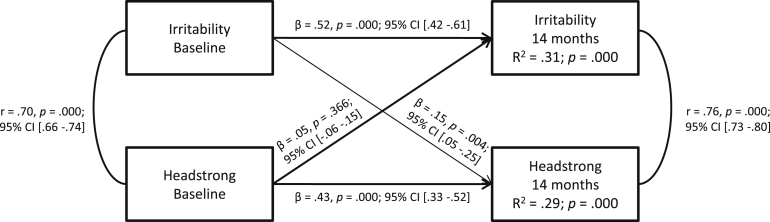
Path analyses of the relation between irritability and headstrong dimensions across time.

Also, the within-domain stability was significantly stronger than the across-domain stability: irritability at baseline was a better predictor of irritability at 14 months (β = 0.52 [95% CI = 0.42–0.61]; *p* ≤ .001) compared to headstrong behaviors at 14 months (β = 0.15 [95% CI = 0.05–0.25]; *p* = .004), whereas the headstrong behaviors dimension at baseline was a significantly better predictor of headstrong behaviors at 14 months (β = 0.43 [95% CI = 0.33−.52]; *p* ≤ .001) than irritability at 14 months (β = 0.05 [95% CI = −0.06–0.15]; *p* = .366). This supports the idea that these constructs are distinct from each other (for more details on the path analytical model, see Figure S1, available online).

Irritability was a significantly stronger predictor than headstrong behaviors for the Internalizing Scale at baseline (irritability: β = 0.43 [95% CI = 0.32–0.54] versus headstrong behaviors: β = 0.06 [95% CI = –0.04–0.17]) as well as at the end of treatment (irritability: β = 0.35 [95% CI = 0.23–0.47] versus headstrong behaviors: β = −0.03 [95% CI = –0.15–0.08]). Conversely, the headstrong behaviors measure was a significantly stronger predictor of the Externalizing Scale at baseline (irritability: β = 0.29 [95% CI = 0.21–0.38] versus headstrong behaviors: β = 0.47 [95% CI = 0.38–0.55]) but not at the end of treatment (irritability: β = 0.22 [95% CI = 0.10–0.33] versus headstrong behaviors: β = 0.29 [95% CI = 0.18–0.40]) (see Table S1, available online, for more details).

Finally, irritability and headstrong behaviors each contributed independently to impairment (irritability: β = 0.24, *p* < .001; headstrong behaviors: β = 0.30, *p* ≤ .001).

### Aim II: To Test Whether ADHD Treatments Are Effective at Treating Irritability

**Figure 1:**
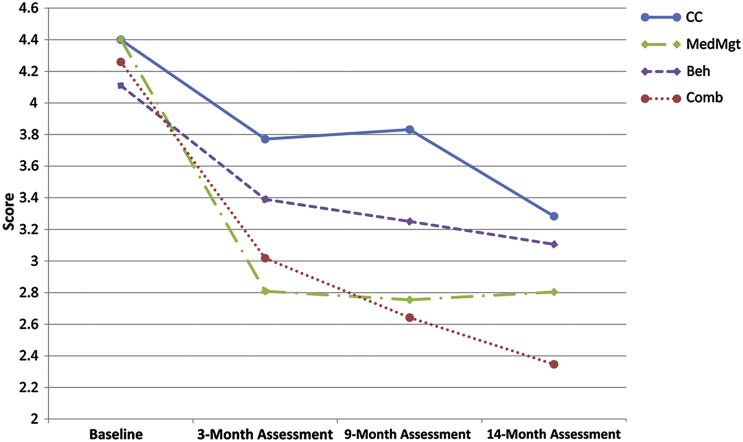
Parent-reported irritability response to multimodal treatment in the 4 treatment groups. Note: Beh = Behavioral treatment; CC = Community Comparison; Comb = Combined treatment; MedMgt = Medication management.

There were no differences in the level of parent-reported irritability at baseline in the 4 treatment groups (F = 0.43, df = 3, *p* = .729). As shown in Figure 1, overall, irritability scores decreased over the course of the treatment. Means and standard deviations at baseline and at 14 months, as well as pre–post effect sizes for each treatment arm, can be found in Table 1. The highest effect size corresponded to combined treatment (0.82), followed by medication management (0.63), community comparison (0.48), and behavioral treatment (0.42).

The random-effects regression model included time, treatment group, and site as predictors of change in irritability. Omnibus Wald tests for the effect of site did not reach significance (site: χ^2^ [5] = 10.61, *p* = .060; site×treatment: χ^2^ [15] = 18.84; *p* = .221); the variable was therefore excluded in a subsequent, more parsimonious model, which included time and treatment group only as predictors of change (Table 2). Effects of time were significant, indicating that irritability scores decreased significantly over time. Effects of treatment group did not reach significance, indicating that irritability scores in the 4 groups were not significantly different across groups. The interaction time×treatment group was significant, indicating that irritability scores changed differentially according to treatment groups. Decomposition of this interaction (Table 2) indicated that from the baseline to the end of the treatment (14-month assessment), the MedMgt group were more likely to improve than Beh (coefficient = 0.58; *p* = .021), but not the CC group (coefficient = −0.42; *p* = .095). Those in the Comb group were more likely to improve than those in the Beh (coefficient = −0.87; *p* = .000) and CC (coefficient = −0.71; *p* = .004) groups, but there was no difference between the MedMgt only and Comb (coefficient = −0.29; *p* = .249). It is important to note that the effect sizes of difference between treatments are more modest compared to the effect sizes between pre- and posttreatment for each group. For example, the effect size of the difference between the Comb and CC arms is 0.34, whereas the difference between CC and Beh is −0.05. However, it is worth noting that the magnitude of the difference between ADHD treatments is best accounted for by the random-effects regression model which, unlike the effect sizes, takes into account the heterogeneity across individuals in their responses over time. Effect sizes are displayed in Table 1.

### Aim III: To Test Whether Irritability Moderates Treatment Response of Children With ADHD

**Figure 2:**
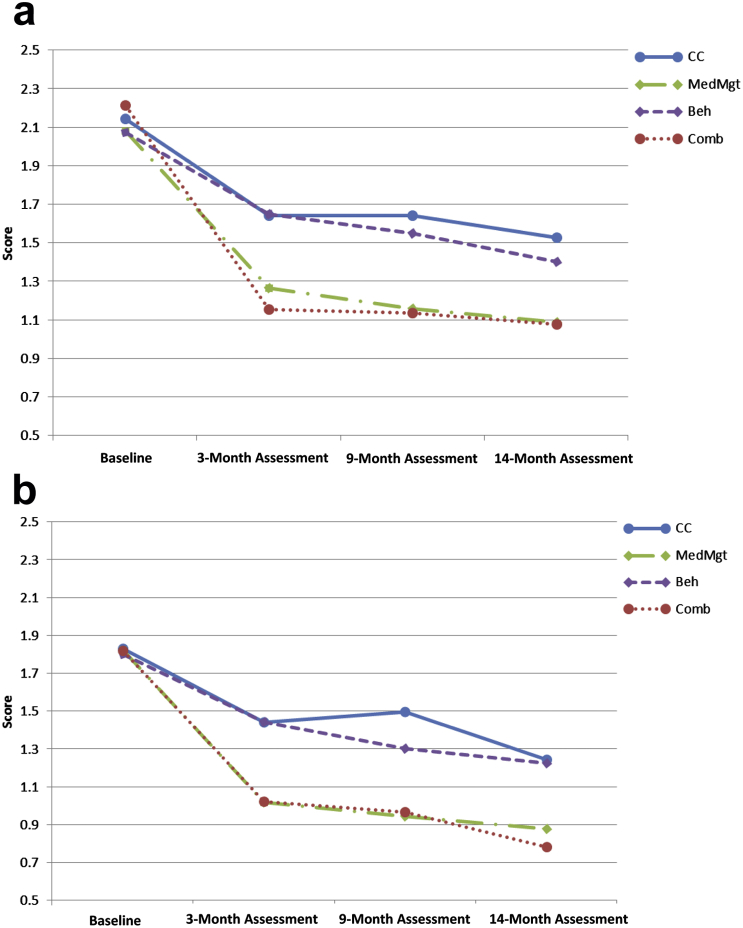
Changes in parent-reported attention-deficit/hyperactivity disorder (ADHD) scores in the 4 treatment groups in individuals with high (a) and low (b) irritability. Note: The categorical outcome was generated using a median split into high and low irritability and is used in this figure for purposes of illustration. However, a dimensional irritability variable is used in the statistical models presented in the text. Beh = behavioral treatment; CC = community comparison; Comb = combined treatment; MedMgt = Medication management.

Irritability (displayed in Figure 2 as a categorical variable using a median split into high and low irritability for illustration but analyzed dimensionally) did not have a differential effect on the reduction in parent-reported ADHD symptoms across the treatment groups across time. Using the dimensional irritability variable, we formally tested this in a random-effects regression model that included baseline irritability, treatment group, time, and site, with the interactions irritability×time, irritability×treatment, time×treatment, and site×treatment (Table 3). Omnibus Wald tests for the effect of site did not reach significance (site: χ^2^ [5] = 8.29, *p* = .141; site×treatment: χ^2^ [15] = 14.15; *p* = .514), for which the variable was excluded in a subsequent, more parsimonious model, which included time, treatment group, and irritability only as predictors of change (Table 3). In addition, we estimated a 3-way interaction model including time×treatment group×irritability for ADHD symptoms as the outcome. As expected, results of the 3-way interaction were not significant (coefficients ranging from −0.02 to 0.01; *p* values ranging from .481 to .898; full results of this model are presented in Table S2, available online). Moreover, the first, more simplified model also presented a better fit compared to the model including the 3-way interaction (AIC = 2924.95 versus 2938.69; BIC = 3064.30 versus 3128.21, respectively).

There was a significant main effect of irritability (reflecting the higher baseline scores of ADHD symptoms in children with high baseline irritability) and a main effect of time (indicating that ADHD scores decreased significantly over time). The effect of site (but not of the interaction site×treatment) was also significant. As shown in Table 3, the interaction treatment×time reached significance. Decomposition of this interaction showed a better response to treatment for children in the MedMgt and Comb arms compared to those in CC or Beh groups, the same as in the original ITT report.^2^

Additional analyses looked at teacher-rated ADHD as an outcome in the previous model. This was to ensure that the paper's main findings held across informant sources, but also because teachers were more likely to be blinded to the treatment condition. As in the case of the parent-rated ADHD, the variable site did not reach significance (site: χ^2^ [5] = 5.33, *p* = .377; site×treatment: χ^2^ [15] = 12.54; *p* = .638) and therefore was dropped from the model. As can be seen in the lower part of Table 3, results were very similar to those using the parent-rated ADHD as an outcome, except for the fact that the main effect of irritability did not reach significance in this case. As in the case of the parent-reported ADHD, results of the 3-way interaction time×treatment group×irritability were not significant for the teacher report (see Table S3, available online).

## Discussion

This study used the MTA data to examine irritability in children with ADHD and its response to different treatments. Our findings were in line with our initial hypothesis that, in children with ADHD, irritability is a separable dimension within the ODD construct. A number of previous studies converge in showing that oppositionality is best thought of as comprising 2 (irritable and headstrong)^17,36^ or 3 (irritable, headstrong, and hurtful)^14-16,37^ dimensions with distinct correlates. We did not have enough items to examine the hurtful dimension in this study, although a previous study suggests that this does exist in children with ADHD.^38^ Consistent with previous studies, irritability in this sample contributed to impairment, was more associated with emotional than conduct problems, and showed longitudinal continuity. Thus, irritability in ADHD has the same pattern of multivariate structure and correlates as in children without ADHD. This lends support to the notion that, instead of irritability being an ADHD-specific phenomenon, it is a dimension that cuts across psychopathology in the manner of the Research Domain Criteria (RDoC) conceptualization.^39^ However, further studies will be required to determine whether the etiological mechanisms underlying irritability differ between individuals with and without ADHD.

Our second aim was to test the hypothesis that symptoms of irritability would diminish with medication. We found that irritability levels decreased in all treatment arms after 14 months. However, the magnitude of the effect sizes for the irritability response to treatment was approximately half of the magnitude for ADHD symptoms in the original study.^2^ In support of our hypothesis, MedMgt was significantly better in reducing irritability than Beh treatment. Surprisingly, MedMgt was not significantly better than CC in reducing irritability (*p* = .095). However, combining MedMgt with Beh treatment was superior to both the Beh treatment alone and the CC intervention, but not compared to MedMgt alone. Beh treatment was not significantly different from the community care intervention. These results show a partial overlap with previous MTA findings regarding other disruptive symptoms. Jensen *et al.*^40^ analyzed the response to treatment of oppositional and aggressive behaviors and found that not only the combined treatment, but also MedMgt and Beh treatments alone, were each superior to the CC intervention. Moreover, in that study there were no differences among the 3 active treatments for oppositional/aggressive symptoms except for the fact that the Comb was superior to the Beh intervention.^40^ This treatment response difference between irritable and oppositional/aggressive behaviors further suggests that it is important to distinguish between these domains. In light of our findings, it is a possibility that, in the case of irritability, combining medication with behavioral treatment confers advantages given the superiority of the combination, but not MedMgt alone, over the CC, although our results did not actually show superiority of the Comb over MedMgt. Also, our study results indicate that not all irritability remits after standard treatment. Whether adjunct treatments, for example those that have been shown to be effective in treating aggression in ADHD,^41^ should be considered for irritability remains to be established.

Finally, we wanted to examine whether high levels of irritability diminished ADHD treatment response. We found that the combined and medication-only treatment arms were superior to the behavioral treatment and the community care interventions at reducing ADHD symptoms, regardless of the level of irritability. This is in keeping with previous reports on the MTA data showing that the comorbidity of ADHD with ODD or CD rarely interacted with treatment response or outcomes.^10,42^

Limitations of this study include the fact that the MTA was not originally designed to examine irritability in children with ADHD, and therefore patient randomization was not stratified by irritability status. Second, the parents in the MTA study were not blinded to treatment group assignments. As such, the extent to which differential outcomes as a function of treatment group were influenced by parental expectations cannot be determined. However, teacher reports of ADHD symptoms were also used as outcomes, and the results were similar to those obtained when using the parent reports. Therefore, considering that teachers were probably blinded (i.e., unlikely to be aware of the treatment allocation),^23^ it is unlikely that parental expectations played a relevant role in the teacher-rated results, although it is possible that parental expectations affected the child’s behavior in a way that carried over into school. Third, it may well be that we were underpowered to detect a 3-way interaction (irritability×time×treatment group) in our models. However, based on previous simulation results, we have estimated that the sample size required to detect differences among the groups in such a 3-way interaction would be more than 7,000 participants, which is unrealistic for most clinical trials.^43,44^ On the other hand, it is reassuring that the graphs shown in Figure 2 did not suggest the presence of this interaction, and if the moderating effect is so small, it is unlikely to be clinically meaningful. Finally, it is worth noting that the community care arm in the MTA study presented with high medication levels (>70% of children were taking medication for ADHD, albeit with much less consistency/monitoring and lower dosing than the MTA-medicated children) and therefore comparisons against this group should be interpreted with caution.

These results have 2 important clinical implications. First, stimulants—a commonly used and relatively safe class of drugs for ADHD—are also helpful for improving irritability in children with ADHD. Moreover, the combination of stimulants and behavioral treatment could help reduce these symptoms further. Second, irritability symptoms did not have a negative effect on ADHD treatment outcomes. Clinicians can proceed with confidence that ADHD treatments will be effective even in the presence of irritability. These 2 aspects had not been demonstrated in major randomized controlled trials in the field and have long remained an area of clinical uncertainty. Our results may also have etiological implications. Based on the fact that irritability improves with treatments that are effective for ADHD symptoms, it would be tempting to assume that common pathophysiology underlies the overlap between irritability and ADHD. Further research should therefore explore this possibility. Also, future studies should investigate whether ADHD treatment in children with irritable symptoms has a beneficial impact on mood symptoms in the medium-to-long term, given the links between irritability and mood disorders.^45^

**Clinical Guidance**

• Irritability is a separable dimension within the ODD construct in children with ADHD.
• Standard ADHD treatments are helpful for reducing irritability in children with ADHD.
• Irritability symptoms do not seem to influence ADHD treatment outcomes. Clinicians can proceed with confidence that ADHD treatments will be effective even in the presence of irritability.

## Figures and Tables

**Table S1:** Linear Regressions With the Irritability and Headstrong Dimensions as Predictors and the Child Behavior Checklist (CBCL) Subscales as Outcomes

Outcome		Predictors								
		Irritability Dimension			Headstrong Dimension			Coefficient	95% CI	Difference Between Predictors (*p*)
CBCL Subscales		β	95% CI	*p*	β	95% CI	*p*			
Withdrawn	Baseline	**0.32**	**(0.21 to 0.43)**	**.009**	0.04	(−0.07 to 0.16)	.438	**−0.27**	**(−0.48 to 0.07)**	**.009**
	14 mo	**0.25**	**(0.13 to 0.37)**	**.027**	−0.00	(−0.12 to 0.12)	.958	**−0.25**	**(−0.48 to 0.03)**	**.027**
Somatic Complaints	Baseline	0.16	(0.04 to 0.27)	.678	0.11	(−0.00 to 0.23)	.059	−0.04	(−0.26 to 0.17)	.678
	14 mo	0.18	(0.06 to 0.31)	.087	−0.01	(−0.14 to 0.11)	.807	−0.20	(−0.43 to 0.03)	.087
Anxious/Depressed	Baseline	**0.49**	**(0.38 to 0.59)**	**.000**	0.01	(−0.09 to 0.11)	.841	**−0.48**	**(−0.66** to **−0.28)**	**.000**
	14 mo	**0.38**	**(0.26 to 0.50)**	**.000**	−0.05	(−0.17 to 0.07)	.405	**−0.43**	**(−0.65** to **0.21)**	**.000**
Social Problems	Baseline	0.17	(0.06 to 0.28)	.652	**0.22**	**(0.11 to 0.33)**	**.000**	0.05	(−0.15 to 0.25)	.652
	14 mo	0.12	(−0.00 to 0.24)	.604	0.06	(−0.06 to 0.18)	.348	−0.06	(−0.29 to 0.17)	.604
Thought Problems	Baseline	0.18	(0.07 to 0.30)	.484	0.11	(−0.01 to 0.22)	.071	−0.08	(−0.29 to 0.14)	.484
	14 mo	0.10	(−0.02 to 0.22)	.718	0.06	(−0.06 to 0.18)	.335	−0.04	(−0.27 to 0.18)	.718
Attention Problems	Baseline	0.19	(0.07 to 0.30)	.603	**0.13**	**(0.02 to 0.24)**	**.024**	−0.05	(−0.26 to 0.15)	.603
	14 mo	0.15	(0.03 to 0.28)	.202	0.01	(−0.12 to 0.13)	.912	−0.15	(−0.38 to 0.08)	.202
Delinquent Behavior	Baseline	**0.10**	**(0.00 to 0.20)**	**.000**	**0.44**	**(0.34 to 0.54)**	**.000**	**0.34**	**(0.16** to **0.53)**	**.000**
	14 mo	0.10	(−0.01 to 0.22)	.100	**0.28**	**(0.17 to 0.40)**	**.000**	0.18	(−0.03 to 0.39)	.100
Aggressive Behavior	Baseline	0.34	(0.25 to 0.42)	.274	**0.42**	**(0.34 to 0.51)**	**.000**	0.08	(−0.07 to 0.24)	.274
	14 mo	0.24	(0.12 to 0.35)	.738	**0.27**	**(0.16 to 0.38)**	**.000**	0.03	(−0.17 to 0.24)	.738
Sex Problems	Baseline	0.06	(0.06 to 0.30)	.289	**0.18**	**(0.06 to 0.30)**	**.003**	0.12	(−0.10 to 0.33)	.289
	14 mo	0.03	(−0.09 to 0.15)	.617	0.09	(0.03 to 0.21)	.139	0.06	(−0.17 to 0.27)	.617
Internalizing Scale^a^	Baseline	**0.43**	**(0.32 to 0.54)**	**.000**	0.06	(−0.04 to 0.17)	.244	**0.37**	**(−0.56** to **−0.17)**	**.000**
	14 mo	**0.35**	**(0.23 to 0.47)**	**.001**	−0.03	(−0.15 to 0.08)	.575	**−0.39**	**(−0.61** to **−0.17)**	**.001**
Externalizing Scale^a^	Baseline	0.29	(0.21 to 0.38)	.026	**0.47**	**(0.38 to 0.55)**	**.000**	**0.17**	**(0.02** to **0.32)**	**.026**
	14 mo	0.22	(0.10 to 0.33)	.456	**0.29**	**(0.18 to 0.40)**	**.000**	0.07	(−0.13 to 0.28)	.456
Total Score	Baseline	0.35	(0.26 to 0.44)	.802	**0.33**	**(0.23 to 0.42)**	**.000**	−0.02	(−0.19 to 0.15)	.802
	14 mo	0.28	(0.16 to 0.39)	.199	**0.14**	**(0.02 to 0.25)**	**.020**	−0.14	(−0.35 to 0.07)	.199

Note: Significant results are shown in boldface.

a The Internalizing Scale is composed of the subscales Withdrawn, Somatic Complaints, and Anxious/Depressed Behavior. The Externalizing Scale is composed of the Delinquent Behavior and Aggressive Behavior scales.

**Table S2:** Parent-Reported Attention-Deficit/Hyperactivity Disorder (ADHD) Response to Multimodal Treatments Including Baseline Irritability as a Factor (3-Way Interaction Model: Irritability×Time×Treatment Group)

Outcome: Parent-Reported ADHD	χ^2^	Coefficient	CI	*p*
Omnibus Tests				
Time	**30.54**	**—**	**—**	**.000**
Treatment Group	0.43	—	—	.934
Irritability	**11.53**	**—**	**—**	**.001**
Time×Treatment Group	**20.51**	**—**	**—**	**.015**
Time×Irritability	1.45	—	—	.695
Irritability×Treatment Group	0.90	—	—	.825
Time×Irritability×Treatment Group	4.27	—	—	.893
Decomposing Time×Treatment Group Interaction				
CC vs. MedMgt	**—**	**−0.38**	**(−0.67** to **−0.09)**	**.011**
CC vs. Beh	—	0.01	(−0.28 to 0.29)	.969
CC vs. Comb	**—**	**−0.41**	**(−0.71** to **−0.12)**	**.006**
MedMgt vs. Beh	**—**	**0.43**	**(0.14** to **0.74)**	**.004**
MedMgt vs. Comb	—	0.06	(−0.25 to 0.37)	.702
Beh vs. Comb	**—**	**0.38**	**(0.07** to **0.69)**	**.016**
Decomposing Irritability×Time×Treatment Group Interaction				
CC vs. MedMgt	**—**	−0.00	(−0.06 to 0.05)	.877
CC vs. Beh	—	−0.01	(−0.07 to 0.05)	.784
CC vs. Comb	**—**	−0.02	(−0.08 to 0.04)	.481
MedMgt vs. Beh	**—**	−0.00	(−0.06 to 0.05)	.898
MedMgt vs. Comb	—	−0.02	(−0.07 to 0.04)	.567
Beh vs. Comb	**—**	0.01	(−0.05 to 0.07)	.663

Note: Significant results are shown in boldface. Beh = behavioral treatment; CC = community comparison; Comb = combined treatment; MedMgt = medication management.

**Table S3:** Teacher-Reported Attention-Deficit/Hyperactivity Disorder (ADHD) Response to Multimodal Treatments Including Baseline Irritability as a Factor (3-Way Interaction Model: Irritability×Time×Treatment Group)

Outcome: Teacher-Reported ADHD	χ^2^	Coefficient	CI	*p*
Omnibus Tests				
Time	**39.91**	**—**	**—**	**.000**
Treatment Group	2.38	—	—	.497
Irritability	1.12	—	—	.291
Time×Treatment Group	15.84	—	—	.070
Time×Irritability	2.11	—	—	.551
Irritability×Treatment Group	1.19	—	—	.754
Time×Irritability×Treatment Group	6.51	—	—	.688
Decomposing Time×Treatment Group Interaction				
CC vs. MedMgt	**—**	**−0.55**	**(−0.91** to **−0.18)**	**.003**
CC vs. Beh	—	−0.24	(−0.61 to 0.13)	.198
CC vs. Comb	—	−0.30	(−0.68 to 0.07)	.111
MedMgt vs. Beh	—	0.30	(−0.05 to 0.66)	.092
MedMgt vs. Comb	—	0.24	(−0.12 to 0.60)	.185
Beh vs. Comb	—	0.06	(−0.05 to 0.66)	.739
Decomposing Irritability×Time×Treatment Group Interaction				
CC vs. MedMgt	**—**	0.02	(−0.05 to 0.09)	.644
CC vs. Beh	—	0.02	(−0.05 to 0.10)	.535
CC vs. Comb	**—**	−0.01	(−0.09 to 0.06)	.742
MedMgt vs. Beh	**—**	−0.01	(−0.07 to 0.08)	.853
MedMgt vs. Comb	—	−0.03	(−0.10 to 0.04)	.424
Beh vs. Comb	**—**	0.04	(−0.04 to 0.11)	.346

Note: Significant results are shown in boldface. Beh = behavioral treatment; CC = community comparison; Comb = combined treatment; MedMgt = medication management.

**Table 1:** Irritability Response to Attention-Deficit/Hyperactivity Disorder (ADHD) Treatment in the 4 Treatment Groups

	Irritability Scores (SNAP Parent Report)								
	Baseline		3-Month Assessment		9-Month Assessment		14-Month Assessment		Within-Group Effect Size (Baseline to 14-Month Assessment)
Treatment Group	Mean	SD	Mean	SD	Mean	SD	Mean	SD	
Community comparison	4.40	2.43	3.77	2.53	3.83	2.56	3.28	2.18	0.48
Medication management	4.40	2.65	2.81	2.37	2.75	2.26	2.80	2.39	0.63
Behavioral treatment	4.11	2.44	3.39	2.31	3.25	2.20	3.11	2.41	0.42
Combined treatment	4.26	2.41	3.02	2.05	2.64	2.08	2.35	2.22	0.82

Treatment Group Comparison									Between-Group Effect Size (Baseline to 14-Month Assessment)
Medication management vs. Community comparison									0.20
Behavioral treatment vs. Community comparison									−0.05
Combined treatment vs. Community comparison									0.34
Medication management vs. Behavioral treatment									.24
Combined treatment vs. Medication management									.13
Combined treatment vs. Behavioral treatment									0.38

Note: SNAP = Swanson, Nolan, and Pelham rating scale.

**Table 2:** Irritability Response to Attention-Deficit/Hyperactivity Disorder Treatment

Outcome: Parent-Reported Irritability	χ^2^	Coefficient	CI	*P*
Omnibus Tests				
Time	**44.52**	**—**	**—**	**.000**
Treatment Group	1.38	—	—	.711
Time×Treatment Group	**33.66**	**—**	**—**	**.000**
Decomposing Time×Treatment Group Interaction				
CC vs. MedMgt	—	−0.42	(−0.92 to 0.07)	.095
CC vs. Beh	—	0.16	(−0.33 to 0.65)	.515
CC vs. Comb	**—**	−**0.71**	**(**−**1.20** to −**0.22)**	**.004**
MedMgt vs. Beh	**—**	**0.58**	**(0.09** to **1.01)**	**.021**
MedMgt vs. Comb	—	−0.29	(−0.78 to 0.20)	.249
Beh vs. Comb	**—**	**0.87**	**(0.39** to **1.36)**	**.000**

Note: Significant results are shown in boldface. Beh = behavioral treatment; CC = community comparison; Comb = combined treatment; MedMgt = Medication management.

**Table 3:** Attention-Deficit/Hyperactivity Disorder (ADHD) Response to Multimodal Treatments Including Baseline Irritability as a Factor

Outcome: Parent-Reported ADHD	χ^2^	Coefficient	CI	*p*
Omnibus Tests				
Time	**57.90**	**—**	**—**	**.000**
Treatment Group	0.19	—	—	.979
Irritability	**21.06**	**—**	**—**	**.000**
Time×Treatment Group	**123.41**	**—**	**—**	**.000**
Time×Irritability	7.50	—	—	.058
Irritability×Treatment Group	0.16	—	—	.984
Decomposing Time×Treatment Group Interaction				
CC vs. MedMgt	**—**	**−0.40**	**(−0.54** to **−0.25)**	**.000**
CC vs. Beh	—	**−**0.03	(−0.17 to 0.11)	.681
CC vs. Comb	**—**	**−0.50**	**(−0.65** to **−0.36)**	**.000**
MedMgt vs. Beh	**—**	**0.37**	**(0.22** to **0.51)**	**.000**
MedMgt vs. Comb	—	**−**0.11	(−0.25 to 0.03)	.136
Beh vs. Comb	**—**	**0.48**	**(0.33** to **0.62)**	**.000**

Outcome: Teacher-Reported ADHD	χ^2^	Coefficient	CI	*p*
Omnibus Tests				
Time	**87.06**	**—**	**—**	**.000**
Treatment Group	1.17	—	—	.620
Irritability	1.05	—	—	.305
Time×Treatment Group	**70.13**	**—**	**—**	**.000**
Time×Irritability	3.10	—	—	.377
Irritability×Treatment Group	1.65	—	—	.648
Decomposing Time×Treatment Group Interaction				
CC vs. MedMgt	**—**	**−0.47**	**(−0.65** to **−0.29)**	**.000**
CC vs. Beh	—	−0.14	(−0.32 to 0.04)	.125
CC vs. Comb	**—**	**−0.35**	**(−0.53** to **−0.18)**	**.000**
MedMgt vs. Beh	**—**	**0.33**	**(0.15** to **0.51)**	**.000**
MedMgt vs. Comb	—	0.12	(−0.06 to 0.30)	.204
Beh vs. Comb	**—**	**0.21**	**(0.03** to **0.39)**	**.022**

Note: Significant results are shown in boldface. Beh = behavioral treatment; CC = community comparison; Comb = combined treatment; MedMgt = Medication management.
